# Exopolysaccharide-Based Bioflocculant Matrix of *Azotobacter chroococcum* XU1 for Synthesis of AgCl Nanoparticles and Its Application as a Novel Biocidal Nanobiomaterial

**DOI:** 10.3390/ma9070528

**Published:** 2016-06-29

**Authors:** Bakhtiyor A. Rasulov, Parhat Rozi, Mohichehra A. Pattaeva, Abulimiti Yili, Haji Akber Aisa

**Affiliations:** 1 Institute of Genetics and Plant Experimental Biology, Uzbekistan Academy of Sciences, Yukori Yuz, Kybray District 111226, Uzbekistan; bakhtiyor_1980@mail.ru; 2 Key Laboratory of Plant Resources and Chemistry in Arid Region, Xinjiang Technical Institute of Chemistry and Physics, Chinese Academy of Sciences, Urumqi 830011, China; parhatruzi@126.com (P.R.); abu@ms.xjb.ac.cn (A.Y.); 3 University of Chinese Academy of Sciences, Beijing 100039, China; 4 Institute of Microbiology, Uzbekistan Academy of Sciences, Tashkent 100128, Uzbekistan; maqsudaziz@mail.ru

**Keywords:** exopolysaccharide-based bioflocculant, AgCl nanoparticles, *Azotobacter chroococcum*, antimicrobial activity

## Abstract

A simple and green method was developed for the biosynthesis of AgCl nanoparticles, free from Ag nanoparticles, using the exopolysaccharide-based bioflocculant of nitrogen fixing *Azotobacter chroococcum* XU1 strain. AgCl nanoparticles were characterized by UV-Vis, X-ray diffraction (XRD), Fourier Transform-Infra Red (FT-IR) and Scanning electron microscopy-energy dispersive X-ray (SEM-EDX). The concentration-dependent and controllable method for the synthesis of AgCl nanoparticles of a certain size and morphology was developed. As-synthesized AgCl nanoparticles were characterized bya high content of AgCl and exhibited strong antimicrobial activity towards pathogenic microorganisms such as *E. coli*, *S. aureus* and *C. albicans*. The biofabricated AgCl nanoparticles can be exploited as a promising new biocidalbionanocomposite against pathogenic microorganisms.

## 1. Introduction

Bioflocculants are organic macromolecular substances secreted by a wide variety of microorganisms [1,2,3,4,5,6], and microbial bioflocculants have attracted considerable attention in recent years due to their biodegradable, harmless and negligible secondary pollution [1]. The polysaccharide-based bioflocculants are exploitedfor theirhigh efficiency in disposing of different of chemicals [7,8,9,10]. The polysaccharide macromolecules in bioflocculants contain monomeric units of sugar molecules such as glucose, mannose, fructose, or rhamnose, etc. [11]. The hydroxyl groups of these sugars strongly associate with metal ions leading to the formation of different types of biomaterials. Besides, they allow the control of shape, size and particle dispersion.

Polysaccharide-based bioflocculants easily form a variety of liquid crystals in aqueous solutions and bioflocculant-mediated processes are highly profitable. Polysaccharide-based bioflocculants been reported for the synthesis of silver nanoparticles (AgNPs). Along with AgNPs, silver chloride nanoparticles (AgCl-NPs) have alsoreceived wide interest. Electrochemical, chemical reduction, surfactants, photochemical reduction, ionic liquid microemulsion, ultrasound irradiation, hydrolysis and ion-exchange reactions and thermal decomposition methods were developed for the synthesis of AgNPs and AgCl-NPs [12,13,14,15,16]. In recent years, the synthesis of silver halides (AgX) received considerable attention since they are highly photosensitive semiconductors. They have been extensively used as photosensitizers andsource materials in photographic films and can also be used as metallic silver precursors [17,18,19]. A total “biological” method for the synthesis of face-centered cubic Ag/AgCl-NPs was documented, using the cellulose of a non-pathogenic *Gluconacetobacter xylinum*. As-synthesized nanocomposite exhibited considerable antibacterial activity against *S. aureus* and *E. coli* [20]. Besides, the chitosan oligomers [21], and bacterial [22] and non-bacterial cellulose were also used as a template to synthesize Ag/AgCl-NPs [23]. These nanocomposites also displayed high antibacterial activity [22]. Moreover, a controlled synthesis of AgCl-NPs by the polysaccharide-based bioflocculant of diazotrophic strain *Bradyrhizobium japonicum* 36 was reported recently, where at equal molar concentrations of Ag^+^ and Cl^−^, the polysaccharide-based bioflocculants act as a template to assemble only AgCl-NPs with an average diameter of 10 nm [24].

In this study we report the green and controlled synthesis method of AgCl-NPs using the expolysaccharide-based bioflocculant of diazotrophic nitrogen-fixing bacterial strain *Azotobacter chroococcum* XU1. As-synthesized AgCl-NPs further tested as a biocidal agent against pathogenic microorganisms, namely *Candida albicans*, *Escherichia coli* and *Staphylococcus aureus*.

## 2. Results

### 2.1. Biosynthesis and Characterization of AgCl-NPs

**Figure 1:**
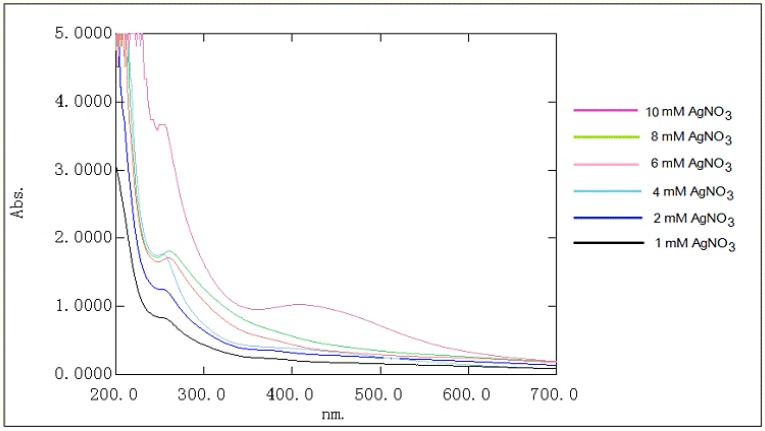
UV-vis spectra of AgCl-NPs in different concentration of AgNO_3_.

Asit was documented, AgCl-NPs develop from a milk-white to a yellowish-brown color in aqueous solution due to excitation of surface plasmon resonance (SPR) [25,26]. Theirsynthesis can be monitored by UV-visible absorption spectra. AgCl-NPs exhibit a broad and strong absorption at 200–350 nm, which can be ascribed to the characteristic absorption of the AgCl semiconductors [27]. The shift in the band may have contributed to the different sizes of the AgCl nanoparticles. Since bioflocculants basically are composed of polysaccharide macromolecules, it is highly possible to reduce the silver ions and stabilize them into the polysaccharide matrix. This evidence makes the parallel synthesis of both AgCl-NPs and AgNPs in the presence of chloride ions and polysaccharide matrix inevitable [21,22,23]. To biofabricate only AgCl-NPs, a concentration-dependent method was evaluated. It was observed that the intensive synthesis of AgCl occurred in low concentrations of Ag^+^ in aqueous solution. The polysaccharide matrix in the reaction mixture enabled the formation of AgCl-NPs with absorption bands at 250–260 nm (Figure 1). This resulted in the absence of absorption within 380–550 nm in the visible-light region, which indicates no synthesis of AgNPs. At a high concentration of Ag^+^, as shown in Figure 1, the formation of both AgCl-NPs and AgNPs was observed (Figure 1; absorption band at 260 and 435 nm assigned as a purple chart). The appearance of the surface plasmon resonance (SPR) transition at 435 nm confirms the production of AgNPs [28,29].

It can be concluded that synthesis of AgCl-NPs and AgNPs is concentration-dependent and the formation of nanoparticles is controllable. For the synthesis of only AgCl-NPs, the concentration of chloride ions plays a key role. Their concentration must be always higher than that of Ag ions in the reaction mixture. However, it is necessary to point out that the AgCl nanoparticles are aggregated into large clusters, which prevented an accurate estimation of their morphology and size distributions [30]. An analogical conclusion was made in previous reports onthe controlled synthesis of AgCl-NPs using the bioflocculant of *Bradyrhizobium japonicum* [24]. Authors reported that when the Ag^+^ concentration exceeded that of Cl^−^, the formation of AgCl-NPs/AgNPs was observed, and in the presence of Cl^−^ ions, the synthesis of AgCl and its assembly over the polysaccharide-based bioflocculant occurs first. When the chloride ions were depleted in the reaction mixture, the excess amount of silver ions was reduced and stabilized in the polysaccharide-based bioflocculant, leading the formation of AgCl-NPs/AgNPs [24].

### 2.2. SEM-EDX

**Figure 2:**
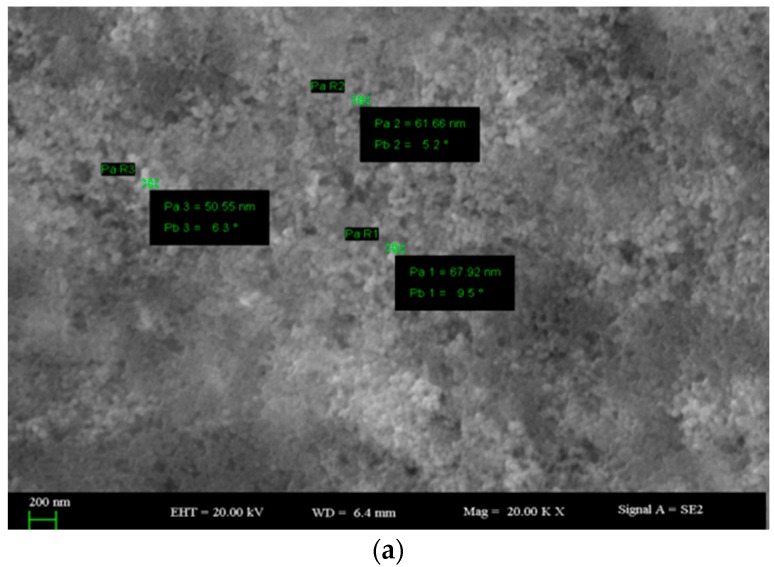

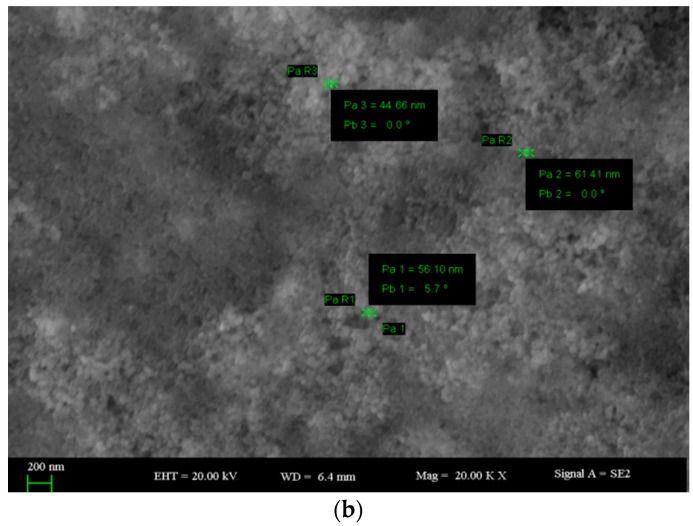
(**a**,**b**) SEM images of AgCl-NPs.

The SEM micrographs of AgCl-NPs clearly showed rough agglomerations of polydisperse character and ranges of approximately 10 to 68 nm (Figure 2).

**Figure 3:**
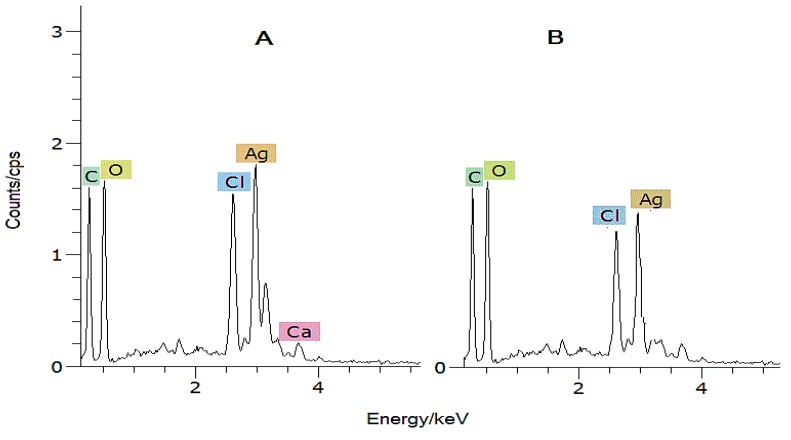
EDX spectra of AgCl-NPs, synthesized using Ag^+^ and Cl^−^ in different concentrations of Ag^+^ ((**A**)—8 mM AgNO_3_; (**B**)—6 mM AgNO_3_).

The Energy dispersive X-ray (EDX) spectra were recorded in order to provide further confirmation on the formation of AgCl-NPs on exopolysaccharide template. The *x*- and *y*-axis labels were energy/keV and count/cps, respectively [30]. The EDX spectra of AgCl-NPs–loaded exopolysaccharide nanocomposite confirm the existence of silver and chlorine atoms. As was seen in the EDX profile, EPS-stabilized AgCl-NPs showed strong signals at ~3 keV for silver atoms, and at ~2.5 keV for chlorine atoms (Figure 3). A semi-quantitative analysis showed the atomic ratio of the Ag and Cl elements was approximately 1:1. This evidence confirms the theoretic stoichiometric atomic ratio between Ag and Cl atoms in AgCl. The data were in agreement with those documented elsewhere [30].

### 2.3. X-ray Diffraction Analysis of AgCl-NPs

**Figure 4:**
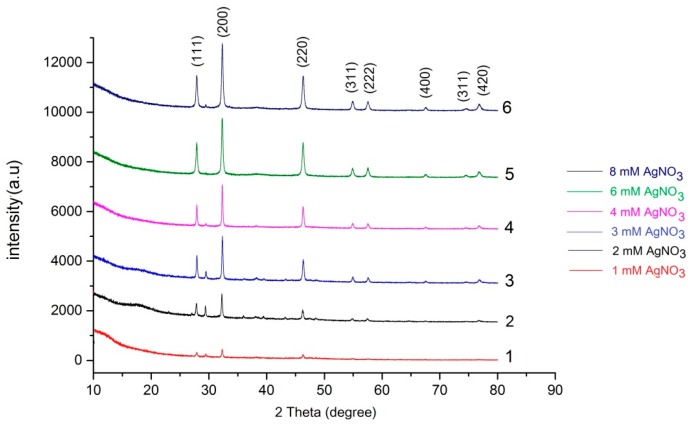
X-ray diffraction (XRD) pattern of AgCl-NPs, synthesized in different concentrations of AgNO_3_.

AgCl-NPs were characterized using the X-ray diffractometer with Cu-Kα radiation operated at a voltage of 40 kV in the scan range of 20 to 80 degree. The XRD pattern of AgCl-NPs exhibited several size-dependent features leading to peak position, height and width. The XRD patterns of AgCl-NPs nanostructures are presented in Figure 4. The distinct diffraction peaks at a 2θ of 27.64°, 32.24°, 46.2°, 54.78°, 57.44°, 67.42°, 74.4° and 76.6° can be attributed to the (111), (200), (220), (311), (222), (400), (331) and (420) planes, which are the typical cubic phase of AgCl crystal (JCPDS file 31-1238) [27,30]. The strong and narrow diffraction peaks in some samples (obtained from relatively high concentrations of Ag^+^ solutions) reveal the highly crystalline structure [27].

Analogical data previously reported by other authors [27,30] suggest the existence of AgCl species in the synthesized nanostructures with the above-mentioned data. Revealed diffraction peaks were sharp and intense, indicating a high degree of crystallinity of the AgCl species.

### 2.4. FT-IR of AgCl-NPs

**Figure 5:**
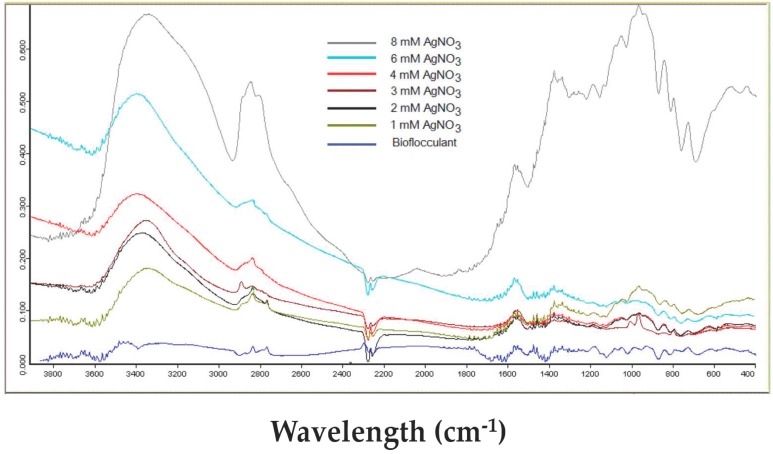
FT-IR spectrum of AgCl-NPs, assembled on the polysaccharide-based bioflocculant of *A. chroococcum* XU1 in different concentrations of Ag^+^.

FT-IR estimation was carried out to investigate the possible functional groups and chemical bonds of AgCl-NPs containing nanobiocomposite. The FT-IR spectra of AgCl-NPs exhibited various characteristic peaks with ranges from 3420 to 800 cm^−1^ (Figure 5). The broadest and strongest peak was observed at 3420 cm^−1^, due to a stretching vibration of the hydroxyl groups (O–H). The broad peaks at 1236–1063 cm^−1^ correspond to the C–O–C stretching from the glycosidic linkages and the O–H bending from alcohols [31,32], whereas the intense peak at 995 cm^−1^ confirms the presence of carbohydrates. Furthermore, weak absorption peaks at 820–972 cm^−1^ were observed, which confirmed that linkages had occurred between monosaccharides in a polysaccharide matrix of the bioflocculant.

In some cases, when the polysaccharide matrix is used for the impregnation of nanoparticles, the absorption maxima of polysaccharides remain unaffected [32,33]. However, when the polysaccharide template is used as a reducing and stabilizing agent, slight changes in the absorption maxima of polysaccharides are observed. Kanmani and Lim (2013) report additional absorption peaks of bacterial exopolysaccharides at 2352 and 1449 cm^−^^1^, which were attributed to the presence of the AgNPs [26]. The biosynthesized AgNPs’ influence on the O−H stretching at 3428 cm^−1^ was also reported [1]. This evidence indicates that Ag/AgCl-NPs’ association with biomolecules is varied due to the nature of the matrix and chemical bonds.

### 2.5. Antimicrobial Activity of AgCl-NPs (the Hole Method)

The antibacterial and antifungal activity of AgCl-NPs, fabricated using the polysaccharide-based bioflocculant of *A. chroococcum* XU1, was investigated against the pathogen bacterial strains *E. coli* ATCC11229, *S. aureus* ATCC6538 and pathogenic fungi *Candida albicans* ATCC1023. The AgCl-NPs samples were tested using the agar well diffusion method and via measuring the radial diameter of inhibition zones. Different concentrations of AgCl-NPs were applied, from 0.1 to 5 mg/mL. The results revealed that all pathogens were inhibited after treatment with AgCl-NPs. The inhibition rate was proportionally dependent on not only the concentration (dilution) of AgCl-NPs, but also on the concentration of the bioflocculant and AgNO_3_, which were used to produce AgCl-NPs. The increase in the AgCl-NPs’ antibacterial activity towards *E. coli* ATCC11229 and *S. aureus* ATCC6538 is shown in Table 1.

The maximum antibacterial activity was observed with 2.5 mg/mL AgCl-NPs and it was several times higher than that of 1 mM of AgNO_3_ applied as a control. Obtained data are in good agreement with those reports, where the dose-dependent impact of Ag/AgCl-NPs was evaluated [20,21,22,23]. The antifungal activity of nanoparticles also widely discussed in the literature and the impact of nanoparticles was evaluated against some genus of fungi, such as *Candida*, *Aspergillus* and *Penicillium* [26]. Some authors reported treatment with low concentrations of AgNPs. Kora and Arunachalam (2011) report the effective suppression of 4 μg /mL AgNPs against *P. aeruginosa* [34], whereas Wei et al. (2012) report 9 μg/mL AgNPs against *B. subtilis* and *E. coli* [35]. Bankura et al. (2012) tested higher concentrations of AgNPs [28]. At a 200 μg/mL concentration of AgNPs, the inhibition zone diameters of *E. coli*, *P. aeruginosa*, *B. cereus*, and *B. subtilis* were 21, 24, 28, and 32 mm, respectively. Similar results were reported by Panacek et al. (2008) [36]. Analogical results were obtained in our previous research, when the AgCl-NP concentrations ranged from 0.25 to 2.5 μg/mL. In these concentrations, the maximal inhibition zones in *C. albicans* ATCC10231 were from 7.5 to 15 mm [24]. The maximum antibacterial activity was observed with 2.5 mg/mL AgCl-NPs and it was 2.5–2.7 times higher than 1 mM AgNO_3_ applied as a control. A similar dose-dependent impact of Ag/AgCl-NPs was also evaluated against *E. coli* and *S. aureus* [20,23].

In our research, the pathogenic strain *C. albicans* ATCC10231 was inhibited by AgCl-NPs, and the antifungal activity proportionally correlated with the concentration of AgCl-NPs applied. With 0.1 mg/mL of AgCl-NPs, the inhibition zone was 7.5 mm; when concentration reached 2.5 mg/mL, the inhibition zone was 15 mm.

## 3. Discussion

Several methods and mechanisms for the green synthesis of AgCl-NPs have been reported. Apart from the application of polysaccharides [20,21,22,23], other reducing agents are also used to reduce Ag^+^ and to synthesize AgCl-NPs. Duran et al. (2014) [37] reported the biogenic synthesis of AgCl-NPs with laccase, whereas Awwad et al. (2015) applied the flowers of *Albizia julibrissin* extract, rich in chlorine ions, as a reducing, chlorinated and capping agent in the formation of Ag/AgCl-NPs [38].

In our research, the exopolysaccharide-based bioflocculant of *A. chroococcum* XU1 was used for the synthesis and stabilization of AgCl-NPs. The exopolysaccharide-based bioflocculant can reduce Ag^+^ from AgNO_3_, and synthesize AgCl-NPs and AgNPs with separate absorption maximums at 250–260 and 435 nm. It was found that the formation of AgCl-NPs and AgNPs was concentration-dependent. In earlier reports, the formation of a mix of AgCl-NPs/AgNPs was documented, since in most cases the concentration-dependent synthesis had not been evaluated [18,19,23,27]. In some reports, chlorine ions were randomly chosen, or controlling theconcentration was not an aim of the surveys [38]. In this report we tried to strictly control the formation of only one type of nanoparticle—AgCl-NP—and the synthesis was carried out atcertain concentrations of Ag^+^ and Cl^−^. At a fixed concentration of the exopolysaccharide-based bioflocculant (11.5 mg/mL) with 1 to 8 mM of Ag^+^ (at the same concentrations of Cl^−^), the enhanced synthesis of AgCl-NPs was observed. When the Ag^+^ concentration exceeded that of Cl^−^, the formation of AgCl-NPs/AgNPs was observed. It is supposed that in the presence of Cl^−^ ions, the synthesis of AgCl and its assembly over the exopolysaccharide-based bioflocculant occurs first. When the chloride ions were depleted in the reaction mixture, an excess amount of silver ions was reduced and stabilized in the exopolysaccharide-based bioflocculant, leading to the formation of AgCl-NPs/AgNPs. Concentration dependent and controlled synthesis of AgCl-NPs was also reported in our previous study [24]. The polysaccharide-based bioflocculant of *B. japonicum* 36 can reduce Ag^+^ from AgNO_3_, and synthesize AgCl-NPs and AgNPs with separate absorption maximums at 335 and 420 nm. It was found that the formation of AgCl-NPs and AgNPs was concentration-dependent. From 1 to 7 mM Ag^+^, the formation of only AgCl-NPs was observed and it exhibited an absorption band at 335 nm. Further increasing the Ag^+^ concentration led to the formation of both AgCl-NPs (335 nm) and AgNPs (420 nm). When the concentrations of both Ag^+^ and Cl^−^ were the same, the AgCl-NPs were the only product, with an absorption band at 335 nm [24].

The XRD analysis of AgCl-NPs exhibited characteristic peaks at 27.64°, 32.24°, 46.2°, 54.78°, 57.44°, 67.42°, 74.4° and 76.6° which were attributed to planes (111), (200), (220), (311), (222), (400), (331), and (420) of the cubic phase of the AgCl crystal, and are in good agreement with early reports [27,31]. Further SEM-EDX and FT-IR analyses also confirmed the presence of nanoparticles and their interaction with some functional groups. The FT-IR analysis showed some new evidence on the influence of AgCl-NPs on some functional groups. Obtained data varies with those from earlier reports with AgNPs [1,24,26,33,36].

As-synthesized AgCl-NPs were further examined for antimicrobial activity towards *E. coli* ATCC11229, *S. aureus* ATCC6538 and *Candida albicans* ATCC1023, and their high biocidal activity was observed. The antibacterial and antifungal activity of AgCl-NPs was also widely reported and discussed in recent surveys [20,21,22,23,24]. Despite the AgCl-NPs being synthesized via different reduction methods and exploiting a wide range of templates, they exhibited considerable antimicrobial activity toward pathogens, such *E. coli*, *S. aureus* and *C. albicans* [20,21,22,23,24]. It can be concluded from the results that the AgCl nanoparticles biofabricated on the basis of the exopolysaccharide-based bioflocculant of *Azotobacter chroococcum* XU1 can be exploited as a promising new biocidal bionanocomposite against pathogenic microorganisms.

## 4. Materials and Methods

### 4.1. Materials and Microbial Strains

The polysaccharide-based bioflocculant producing diazotrophic strain *A. chroococcum* XU1, and pathogenic strains *Candida albicans* ATCC10231, *Escherichia coli* ATCC11229, *Staphylococcus aureus* ATCC6538 were obtained from the Culture Collection of State Key Laboratory Basis of Xinjiang Indigenous Medicinal Plants Resource Utilization, Xinjiang Technical Institute of Physics and Chemistry, CAS.

All solutions were made of using ultra filtered high purity deionized water. Reagents and chemicals used in this study were of analytical grade.

### 4.2. Production of Bioflocculant

Production and purification of bioflocculant by *A. chroococcum* XU1 was developed in our previous experiments [24,25,39] and its fractionation into protein, low- and high-molar-weight polysaccharides was used as a part of protocol for green synthesis of AgNPs [24,25]. Production of bioflocculant carried out in 2 L flasks containing 1 L of the modified medium with 150 rpm at 30 °C under intensive aeration. The composition of the modified medium was as follows: sucrose, 20 g/L; MgSO_4_·7H_2_O, 0.2 g/L; KH_2_PO_4_, 0.2 g/L; NaCl, 0.1 g/L; CaCO_3_, 10 g/L. The initial pH value of the medium was adjusted to 7.0. Each flask was inoculated with 4% (*v*/*v*) of the seed culture and incubated at 30 °C with shaking at 150 rpm for three days. Samples were withdrawn at different time intervals and monitored for cell growth and flocculating activity. Culture broth was centrifuged at 10,000 rpm for 15 min to separate the cells which were washed twice with distilled water.

### 4.3. Purification of Bioflocculant

Bioflocculant purification was carried out as documented elsewhere [1]. The culture broth was centrifuged at 10,000 rpm for 20 min. To the supernatant were added three volumes of cold ethanol instantly, until white cotton-like flocks were formed. The precipitate centrifuged at 10,000 rpm for 15 min. Then, the bioflocculant dialyzed against de-ionized water at 4 °C overnight to obtain purified bioflocculant, free from minerals.

### 4.4. Synthesis of AgCl-NPs

Preparation of AgCl-NPs was carried by a method, developed by us [24]. Freshly prepared bioflocculant was mixed with 10 mL of 2 mM NaCl solution with a vaccination needle and exposed to 10 mM AgNO_3_ solution. The reaction mixture was left overnight at room temperature. For FT-IR and XRD analysis the reaction solution was centrifuged at 15,000 rpm for 5 min to obtain AgCl-NPs. The prepared AgCl-NPs were washed four times with distilled water to remove the impurities absorbed on the surface of AgCl-NPs.

### 4.5. Characterization of AgCl-NPs

Polysaccharide-based bioflocculant reduction of Ag^+^ ions, formation of AgCl-NPs/AgNPs in aqueous solution was monitored for 12 h, fivedays, 10 days and twomonths by measuring the ultraviolet-visible absorbance spectrum of the solution using a UV-visible spectrophotometer (TU-1901, Beijing Purkinje General Instrument Co., Ltd., Beijing, China) in the range of 200–700 nm [24]. Scanning electronmicroscope EDX analysis of the AgCl-NPs was performed using a Hitachi apparatus, Japan. The phase composition and crystal structure of the AgCl-NPs was determined using XRD (Bruker D8 advance, Karlsruhe, Germany). For this, the dried sample was prepared by placing on the microscopic glass slide and the diffractogram was recorded using Cu-K α radiation and a nickel monochromator filtering wave at a voltage and current of 40 kV and 30 mA, respectively. The FT-IR spectrum of the polysaccharide-based bioflocculant-stabilized AgCl-NPs were analyzed using FT-IR spectroscopy (JASCO FT-IR 460, Daejon, Korea) operated at resolution of 4 cm^−1^. For the measurement of FT-IR spectrum, the dried sample was powdered by grinding with KBr pellets and pressed into a mold. The spectrum was recorded at a frequency range of 500–4000 cm^−1^ [24].

### 4.6. Antibacterial and Antifungal Activities of AgCl-NPs

#### 4.6.1. Incubation of Pathogenic Strains

All bacterial strains were cultured following manufacturers’ culturing guidelines. Typically, *E. coli* ATCC11229 and *S. aureus* ATCC6538 strains were cultured in Luria-Bertani (LB) culture medium (tryptone, 10 g/L; yeast extract, 5 g/L; NaCl, 10 g/L) at 37 °C. In all the experiments, the concentrations of bacteria were determined by optical density at 600 nm.

*C. albicans* ATCC10231 was cultured in culture medium comprising of: glucose, 40 g/L and peptone, 10 g/L [25,39].

#### 4.6.2. The Hole Method

The antibacterial and antifungal activity of the polysaccharide-based bioflocculant stabilized AgCl-NPs was measured using the agar well diffusion method. Bacterial and fungal pathogens such as *E. coli*, *S. aureus* and *C. albicans* were used as indicator strains for this analysis. The bacterial and fungal strains were aseptically inoculated into respective broth, and then incubated at 37 °C. Samples from the culture liquids of respective pathogen plated on Petri plates and wells were made using an agar well borer. Different concentrations of AgCl-NPs were added to these wells, and the plates were incubated at 37 °C for 24 h. Zone of inhibitions were estimated by measuring the diameter of the bacterial growth inhibition zone. The values were averaged from the three independent experiments [25,39].

## 5. Conclusions

In conclusion, the polysaccharide-based bioflocculant of *A. chroococcum* XU1 can reduce Ag^+^ from AgNO_3_, and synthesize AgCl-NPs with absorption maximum at 250–260 nm. It was found that formation of AgCl-NPs and AgNPs was concentration dependent. At equal molar concentrations of Ag^+^ and Cl^−^ of the polysaccharide-based bioflocculant act as a template to assembly only AgCl-NPs with an average diameter 10–68 nm. XRD analysis of AgCl-NPs exhibited characteristic peaks at 27.64°, 32.24°, 46.2°, 54.78°, 57.44°, 67.42°, 74.4° and 76.6° can be attributed to the (111), (200), (220), (311), (222), (400), (331) and (420) of cubic phase of AgCl crystal. When Ag^+^ concentration exceeded than that of Cl^−^ formation of AgCl-NPs/AgNPs observed. It is supposed that in the presence of Cl^−^ ions, synthesis of AgCl and its assembly over the polysaccharide-based bioflocculant occurs first. When the chloride ions depleted in the reaction mixture, excess amount of silver ions are reduced and stabilized in the polysaccharide-based bioflocculant, leading the formation of AgCl-NPs/AgNPs. As-synthesized AgCl-NPs exhibited strong antibacterial and antifungal activity against *E. coli*, *S. aureus* and *C. albicans*.

## Figures and Tables

**Table 1:** Antibacterial activity of AgCl-NPs in 2.5 mg/mL concentration.

Strains	Inhibition Zone, mm
*E. coli* ATCC11229	22
*S. aureus* ATCC6538	23
*C. albicans* ATCC10231	15
